# Supplementary figures and images for: Validation of an Algorithm for Measurement of Sedentary Behaviour in Community-Dwelling Older Adults
Source: Sensors (Basel). 2023 May 9;23(10):4605. doi: 10.3390/s23104605 (PMC10223684; doi:10.3390/s23104605)

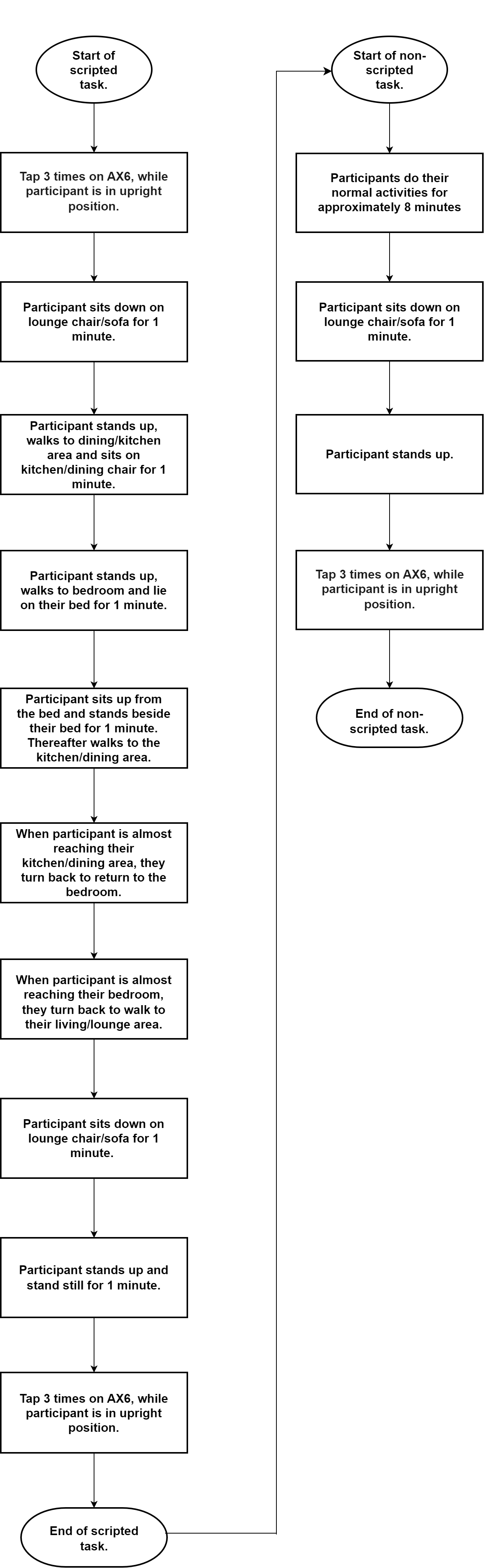

Supplement: Supplementary file 1 [file sensors-23-04605-s001.zip › Figure S1.png]
